# Aging Resistance Improvement of Rubber–Plastic-Based TPE-Modified Asphalt System with Different Plastic Types

**DOI:** 10.3390/ma19204364

**Published:** 2026-10-16

**Authors:** Liuyang Zhang, Keliang Yi, Jianping Xiong, Nana Mao, Peikai He, Peng Xu, Hao Yang, Hongyu Zhou, Yuetan Ma

**Affiliations:** 1 Guangxi Key Laboratory of Road Structure and Materials, Nanning 530007, China; y17770261@163.com (L.Z.); ykljwyx@outlook.com (K.Y.); xjpjwyx@outlook.com (J.X.); 2 School of Traffic and Transportation Engineering, Changsha University of Science & Technology, Changsha 410004, Chinahongyuzhou@csust.edu.cn (H.Z.);; 3 Guangxi Transportation Science and Technology Group Co., Ltd., Nanning 530007, China; 4 Hebei Communications Investment Resource Development and Utilization Co., Ltd., Shijiazhuang 050000, China

**Keywords:** modified bitumen, reclaimed rubber, recycled polyolefins, reactive extrusion, aging resistance, multiscale characterization

## Abstract

**Highlights:**

- Surface activation and reactive extrusion produced rubber–plastic composite modifiers.
- Aging was examined through rheological, chemical, and surface measurements.
- The composite-modified binders showed lower aging indices than the styrene–butadiene–styrene reference.
- The low-density polyethylene composite showed the lowest rheological aging indices among the three composites.
- After pressure and ultraviolet aging, the high-density polyethylene composite had the lowest carbonyl and roughness aging indices among the three composites.

**Abstract:**

This study evaluated the aging resistance of bitumen modified with integrated rubber–plastic thermoplastic elastomers (TPEs). Surface-activated low-Mooney reclaimed rubber was compounded with recycled high-density polyethylene, low-density polyethylene, or polypropylene by twin-screw reactive extrusion using a maleic anhydride-grafted styrenic block copolymer compatibilizer. Reference binders contained styrene–butadiene–styrene (SBS), rubber alone, or individual polyolefins. Aging comprised the rolling thin-film oven test, pressure aging vessel conditioning of oven-aged residues, and ultraviolet exposure. Dynamic shear rheometry, Fourier transform infrared spectroscopy, and atomic force microscopy characterized rheological, chemical, and topographic changes. The largest rutting factor and carbonyl aging indices occurred after sequential oven and pressure aging under the selected conditions. The TPE-modified binders showed lower rutting factor and carbonyl aging indices and smaller relative roughness increases than the SBS reference under each protocol. Among the three TPE formulations, the low-density polyethylene composite had the lowest rutting factor aging indices, whereas the high-density polyethylene composite had the lowest carbonyl aging indices and relative roughness increases after pressure and ultraviolet aging. These findings support the laboratory aging-resistance potential of integrated TPEs, with improved interfacial compatibility providing a plausible explanation. The approach offers a route for the high-value utilization of waste rubber and plastics.

## 1. Introduction

During long-term service, bitumen pavements are inevitably subjected to the multi-field coupled effects of light, heat, oxygen, and traffic loading, leading to the irreversible aging and deterioration of bitumen binders. This subsequently triggers early distresses such as cracking, raveling, and rutting, thereby severely shortening the service life of roads [1,2,3,4]. To enhance the long-term durability of pavement materials, Behnood et al. [5] and Emtiaz et al. [6] extensively investigated polymer modification technologies, pointing out that this technique is currently one of the most effective means to extend the service cycle of pavements. Nevertheless, driven by the escalating demands of heavy-duty transportation and the growing incidence of severe weather phenomena, the durability bottlenecks of traditional single-polymer-modified bitumen in complex environments have become increasingly prominent [7,8,9]. In recent years, Yu et al. [10] proposed converting waste rubber and plastic materials into high-performance bitumen modifiers. This approach not only achieves the high-value recycling of solid wastes but is also regarded as a highly promising green pathway to enhance the long-term durability of bitumen [11,12].

SBS is still regarded as a benchmark modifier in polymer-modified bitumen. However, its molecular structure is vulnerable to aging environments. Chen et al. [13] and Guo et al. [14] reported that the unsaturated bonds in the butadiene phase provide active sites for oxidative and photo-oxidative reactions. Once exposed to heat, oxygen, or UV radiation, these sites can initiate radical-driven degradation, including chain scission and cross-linking, ultimately weakening the aging resistance of SBS-modified bitumen. This results in a significant degradation of its anti-aging performance over service time [15,16]. Aurilio et al. [17] and Tarefder et al. [18] further noted that aging severity can markedly affect the fatigue-cracking resistance and viscoelastic response of SBS-modified bitumen. Jamal et al. [19] pointed out that crumb rubber exhibits a certain shielding effect against UV irradiation. Wang et al. [20] and Zhang et al. [21] found that different rubber-modified systems show markedly different responses to environmental aging. In addition, Ma et al. [22] suggested that waste-plastic modification helps improve the high-temperature stiffness and rutting resistance of bitumen. Liang et al. [23] further elucidated the phase evolution and thermal storage stability of polyethylene-modified bitumen. Studies by Aldagari et al. [24] showed that, although single-plastic-modified systems can improve high-temperature performance, they generally suffer from insufficient compatibility, poor storage stability, inadequate low-temperature toughness, and pronounced performance deterioration after aging. It is therefore evident that neither single-rubber modification nor single-plastic modification can simultaneously satisfy the requirements of high-temperature stability, interfacial compatibility, and long-term aging resistance [25,26].

Based on these considerations, rubber–plastic composite modification has gradually emerged as an important research focus in recent years. According to Ge et al. [27], the co-addition of discarded tire rubber and recycled polyethylene plays a positive role in optimizing the phase interface between the modifying agents and the neat bitumen. Wang et al. [28] achieved the synergistic stabilization of crumb rubber and polyethylene in bitumen through screw extrusion and subsequently proposed a thermoplastic elastomer modification strategy based on reclaimed rubber [29]. Ma et al. [30,31,32] further reported that recycled rubber–plastic composite modifiers outperform some single-component systems in terms of compatibility, thermal storage stability, rheological properties, and resistance to environmental aging. Roja et al. [33] suggested that compatibilizers play an important role in regulating the aging behavior of polyethylene-modified bitumen. Studies by Nisar et al. [34] and Yu et al. [35] on LDPE–SBS composite systems indicated that composite modification shows considerable potential in enhancing rheological stability and preserving microstructure. Nisar et al. [36] further pointed out that, although the aging behavior of LDPE–SBS composite-modified bitumen has attracted increasing attention, its evaluation has still been largely confined to conventional composite systems. Overall, existing studies have mainly focused on direct-blending rubber/plastic systems or LDPE–SBS hybrid systems. In contrast, limited attention has been paid to constructing rubber–plastic-based integrated thermoplastic elastomers (TPEs) from surface-activated low-Mooney reclaimed rubber and recycled polyolefins via reactive extrusion, and subsequently applying them to improve the aging resistance of modified bitumen. In particular, systematic comparisons regarding the distinct roles of HDPE, LDPE, and PP in such composite systems remain lacking.

In addition to the modifier system itself, methods for evaluating aging resistance are evolving from single macroscopic performance tests toward multi-scale synergistic characterization. Chen et al. [37] reported that the combined use of rheological and chemical indices is helpful for characterizing the long-term oxidative aging of composite-modified bitumen. Zheng et al. [38] elucidated the UV aging mechanism of SBS composite systems from the perspective of two-dimensional infrared correlation spectroscopy. Peng et al. [39] showed that the UV aging behavior of waste-polyethylene-modified systems can be identified through changes in functional groups and microstructural evolution. Xie et al. [40] confirmed that integrating rheological tests, FTIR, and microstructural analysis provides a more comprehensive understanding of the aging characteristics of composite-modified bitumen. Wang et al. [41] further showed that MSCR, LAS, and microscopic analysis can provide complementary evidence for aging evaluation of polymer-modified bitumen. Studies by Chen et al. [42], Kumar et al. [43] and Sun et al. [44] also indicated that a single rheological index or chemical index is often insufficient to fully capture the intrinsic anti-aging performance of composite-modified systems. Therefore, a systematic comparison based on the rutting factor aging index, carbonyl aging index, and AFM roughness evolution, together with the establishment of a multi-index comprehensive evaluation framework, is of great significance for clarifying the anti-aging advantages of rubber–plastic-based integrated TPE-modified bitumen.

Based on the above considerations, this study used 70-penetration-grade base bitumen as the matrix. SBS-modified bitumen, crumb-rubber-modified bitumen, LMMR-modified bitumen, and single-polyolefin-modified bitumen were selected as reference binders. Surface-activated LMMR was compounded with HDPE, LDPE, and PP in the presence of SEBS-g-MAH by twin-screw reactive extrusion to prepare rubber–plastic integrated TPEs. The prepared TPEs were then incorporated into base bitumen to obtain the corresponding modified binders. Three aging protocols, namely RTFOT, PAV, and UV aging, were applied to simulate different aging environments. DSR, FTIR, and AFM were used to evaluate the deterioration of high-temperature rheological behavior, the accumulation of oxidative functional groups, and the evolution of surface morphology. A multi-index normalization method was further introduced to compare the overall anti-aging performance of different modified bitumen systems. This study aims to reveal the synergistic role of surface activation and reactive extrusion in enhancing the aging resistance of rubber–plastic integrated TPE-modified bitumen, and to provide a material design basis for the high-value utilization of waste rubber and plastics in durable bitumen pavements.

## 2. Test Materials

### 2.1. Raw Materials

#### 2.1.1. Base Bitumen

Donghai-70# base bitumen obtained from China Petrochemical Group Co., Ltd., Beijing, China, was used as the matrix binder in this work. The measured physical parameters were in accordance with the criteria of JTG F40-2004 [45], with the corresponding results reported in Table 1.

#### 2.1.2. SBS Modifier

A road-grade linear styrene–butadiene–styrene (SBS) modifier was used as the conventional polymer modifier. The material was supplied by Sinopec Baling Petrochemical Co., Ltd., Yueyang, Hunan, China. Its main technical properties are listed in Table 2.

#### 2.1.3. Waste Polymers

Based on the concept of high-value utilization of waste materials, this study introduced ground tire rubber (GTR), low-Mooney reclaimed rubber (LMMR) and recycled waste plastics as composite modifiers. Both GTR and LMMR are provided by Hebei Jiaoke Material Technology Co., Ltd., Shijiazhuang, Hebei, China, and their technical indicators are shown in Table 3.

In addition, three typical recycled plastics were selected in the experiment: high-density polyethylene (HDPE), low-density polyethylene (LDPE) and polypropylene (PP), which were provided by Shanghai Huimei Plastic Co., Ltd., Shanghai, China. The appearance of these three semi-crystalline polymers is flat spherical particles, and their basic thermodynamic and physical parameters are shown in Table 4.

#### 2.1.4. Compatibilizer

Maleic-anhydride-grafted styrene–ethylene/butylene–styrene, abbreviated as SEBS-g-MAH, was selected as the compatibilizer for the rubber–plastic integrated TPE modifier system. The commercial grade was FG1901, supplied by Kraton (The Woodlands, TX, USA). SEBS-g-MAH was introduced to enhance the interfacial interaction between LMMR and recycled polyolefins during reactive extrusion. Its technical properties are listed in Table 5.

### 2.2. Preparation of Rubber–Plastic-Based Elastomer-Modified Bitumen

#### 2.2.1. Preparation Process of Rubber–Plastic-Based TPE

Prior to twin-screw reactive extrusion, LMMR was pre-activated via a proactive mild micro-oxidation strategy. Under the synergistic regulation of catalyst and micro-oxygen atmosphere, waste rubber powder underwent thermo-oxidative devulcanization in a sealed reactor at 180–200 °C, 0.3–0.5 MPa, and an oxygen concentration of 20–30% for 30–40 min. Unlike conventional high-energy shear devulcanization, this method efficiently deconstructed the internal rubber–rubber and carbon black–rubber interaction networks in waste rubber powder without using expensive devulcanizing agents. Meanwhile, moderate cleavage of rubber molecular chains reduced the Mooney viscosity, and reactive oxygen-containing functional groups were introduced in situ onto the particle surface. This surface activation markedly enhanced the sol fraction and chemical reactivity of LMMR, thereby facilitating subsequent homogeneous melt blending and interfacial grafting with polyolefins (HDPE, LDPE, and PP) in the presence of SEBS-g-MAH.

For TPE preparation, the pretreated LMMR and plastic particles were first premixed according to the proportions listed in Table 6 and then fed into a twin-screw extruder (Thermo Fisher Scientific, Waltham, MA, USA). The barrel temperature profile was set to 120 °C in Zone 1, 180 °C in Zone 2, 220 °C in Zones 3–4, 190 °C in Zone 5, and 180 °C in Zone 6. The screw speed and feed rate were fixed at 80 rpm and 15 rpm, respectively, to match the differences in melting temperature and melt-flow characteristics of the components. The rubber/plastic phase was fixed at a mass ratio of 70/30, and SEBS-g-MAH was added at 5 phr relative to the total rubber–plastic phase. The resulting LMMR/plastic/SEBS-g-MAH blend was then reintroduced into the extruder and extruded under constant processing conditions. The extrudates were finally quenched in cold water and pelletized to obtain rubber–plastic-based integrated TPE pellets.

#### 2.2.2. Preparation Process of Rubber–Plastic Elastomer-Modified Bitumen

For bitumen preparation, the base bitumen was first heated to approximately 180 °C. Rubber–plastic-based TPE pellets were then added gradually and premixed for 15 min at 300–500 rpm. The blending system was subsequently subjected to high-speed shearing at 4500–5000 rpm for 30 min. After shearing, the bitumen was further allowed to develop in a high-speed disperser at 300 rpm for 20 min to obtain the target-modified bitumen. In this study, the dosage of rubber–plastic-based TPE was fixed at 15 wt.% of the base bitumen, and the detailed formulations of the modified bitumens are presented in Table 7.

The reference binders were prepared using the same bitumen blending, shearing, and development procedure described above. SEBS-g-MAH was also incorporated into the reference formulations.

## 3. Experimental Methods

**Figure 1:**
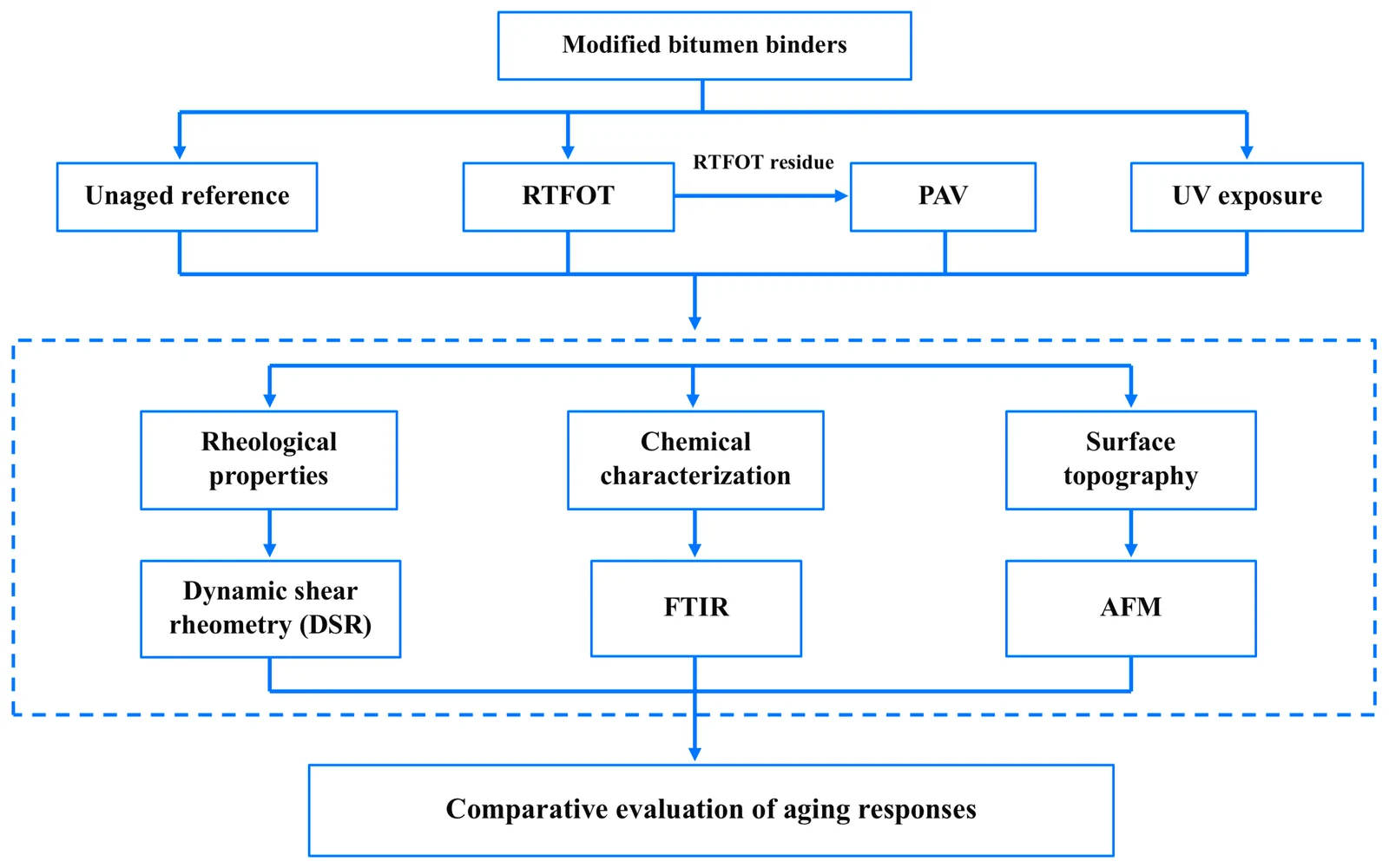
Schematic overview of the preparation, aging, and characterization of rubber–plastic-based TPE-modified bitumen.

HDPE, LDPE, and PP were selected as representative recycled polyolefins to compare plastic-type-dependent responses within a common rubber–plastic TPE formulation. HDPE and LDPE differ in chain branching and crystallinity, features that influence polyethylene phase behavior in bitumen. PP provides a further structural contrast through its pendant methyl groups and, for the material used here, a higher melting point (Table 4). The listed melting points of all three plastics are below the peak barrel setting of 220 °C, supporting their comparison using a common extrusion temperature profile. The rubber/plastic ratio and compatibilizer dosage were held constant to provide a consistent formulation basis for evaluating the rheological, chemical, and morphological aging responses associated with the choice of plastic. The laboratory aging and characterization workflow is illustrated in Figure 1.

### 3.1. Aging Test Protocols

Three laboratory protocols were used to examine short-term thermo-oxidative aging, accelerated long-term thermo-oxidative aging under pressurized air, and UV photo-aging.

#### 3.1.1. RTFOT Short-Term Thermal Oxidation Aging

According to JTG E20-2011 (T0610) [46], (35 ± 0.5) g of modified bitumen binder was subjected to RTFOT aging at (163 ± 0.5) °C, with an airflow rate of (4.0 ± 0.2) L·min^−1^ and a carousel rotation speed of (15 ± 0.2) r·min^−1^ for 85 min. The resulting residue was used for subsequent performance tests and PAV aging.

#### 3.1.2. PAV Long-Term Pressure Thermal Oxidation Aging

According to JTG E20-2011 (T0630-2011), (50 ± 0.5) g of RTFOT-aged binder was poured into a stainless-steel pan to form a bitumen film approximately 3.2 mm thick and subjected to PAV aging at (100 ± 0.5) °C and (2.1 ± 0.1) MPa for 20 h. The aged residue was subsequently vacuum-degassed at 163 °C until no visible bubbles remained and was used for subsequent performance tests.

#### 3.1.3. Ultraviolet Light Aging (UV)

The modified bitumen binder was heated, thoroughly mixed, and uniformly spread on glass plates or stainless-steel pans to form films approximately 1 mm thick. The specimens were continuously irradiated for 120 h in a self-designed UV aging chamber at 25 °C using a high-pressure mercury lamp with a dominant wavelength of 365 nm and an irradiance of 20.0 mW·cm^−2^. The aged specimens were collected for subsequent performance tests.

### 3.2. Performance Testing Methods

#### 3.2.1. DSR Dynamic Shear Rheology Test

A TA Instruments ARES-G2 (TA Instruments, New Castle, DE, USA) dynamic shear rheometer was adopted to characterize the high-temperature viscoelastic behavior of bitumen binders under four different aging states (original unaged, RTFOT short-term thermal oxidation, PAV long-term pressure aging, and UV photoaging). In this measurement system, parallel plates with a diameter of 25 mm were installed, and the testing gap between upper and lower plates was controlled at 1.0 mm. All oscillatory shear tests were carried out under strain-controlled mode, and strain amplitudes were strictly limited within the linear viscoelastic range of bitumen samples to avoid structural damage. The fixed testing temperature was set at 64 °C, alongside a constant angular frequency of 10 rad·s^−1^ (equivalent to 1.59 Hz). After acquiring complex shear modulus (G*) and phase angle (δ) from test outputs, the rutting factor G*/sinδ was further computed. This index was utilized to quantify the anti-rutting capacity of bitumen at high temperature and reflect the stiffness growth caused by aging degradation. This testing scheme conforms to standard bitumen rheology evaluation methods for pavement engineering, aiming to reveal the aging hardening mechanism of composite-modified binders. For each binder formulation and aging condition, the reported rheological values were obtained from a single DSR determination. The rheological aging indices were calculated from the corresponding aged and unaged measurement values.

#### 3.2.2. Fourier Transform Infrared Spectroscopy (FTIR) Test

Chemical structural variations in bitumen binders before and after three aging treatments were analyzed via a Bruker TENSOR (Bruker Optik GmbH, Ettlingen, Germany) Fourier transform infrared spectrometer fitted with an ATR (attenuated total reflectance) testing module. Each FTIR spectrum was acquired by co-adding 32 scans over the wavenumber range of 4000–400 cm^−1^ at a resolution of 4 cm^−1^. These scans were used for signal accumulation and were not treated as independent experimental replicates. To guarantee comparability among all sample groups, unified sample preparation methods and instrument parameter settings were implemented in all spectral tests. The characteristic absorption peak located around 1700 cm^−1^, which corresponds to carbonyl groups, was selected as the core analysis target. It can quantitatively reflect the accumulation of oxidative functional groups generated in the bitumen matrix under thermal oxidation and ultraviolet irradiation.

#### 3.2.3. AFM Atomic Force Microscope Test

An Oxford Instruments Asylum Research MFP-3D Infinity atomic force microscope (Oxford Instruments Asylum Research, Inc., Santa Barbara, CA, USA) was utilized to capture the nanoscale surface morphology of bitumen binders under various aging conditions. Liquid hot bitumen was evenly coated on clean glass substrates to prepare flat test samples. All prepared specimens were placed inside a dust-free chamber and stored at ambient temperature for 24 h, which eliminated residual thermal stress and stabilize surface microstructure to reduce testing error. Tapping mode was chosen for all AFM scanning at room temperature; the scanning field was set to 20 μm × 20 μm, and the scanning speed was fixed at 1.0 Hz. Consistent sample pretreatment steps and scanning parameter configurations were adopted for every bitumen group to ensure the reliability of roughness comparison results. Before extracting root-mean-square roughness R_q_ values, all original topography images were processed with the Flatten function to eliminate interferences, including sample inclination, scanner drift and baseline curvature, so as to obtain the intrinsic surface fluctuation characteristics of bitumen.

The quantitative results are interpreted descriptively. Replicate-based uncertainty estimates and inferential statistical tests are not reported in the present analysis; differences between formulations are therefore discussed as observed trends. The parameters used to evaluate aging and the corresponding calculation equations are presented in Section 4.1, Section 4.2 and Section 4.3.

## 4. Results and Analysis

### 4.1. Rutting Factor Aging Index

To assess the influence of aging on the high-temperature rheological response of modified bitumen binders, the rutting factor aging index, denoted as $AI_{G^{*}/\sin\delta }$, is employed in this study to characterize the relative change in rutting factor before and after aging. It is defined as follows:

(1) $$AI_{\left(G^{*}/\sin\delta \right)}=\frac{{\left(G^{*}/\sin\delta \right)}_{\mathrm{aged}}}{{\left(G^{*}/\sin\delta \right)}_{\mathrm{unaged}}}$$

where ${\left(G^{*}/\sin\delta \right)}_{\mathrm{aged}}$ and ${\left(G^{*}/\sin\delta \right)}_{\mathrm{unaged}}$ represent the rutting factors of the aged and unaged bitumen binders, respectively. Generally, a higher $AI_{\left(G^{*}/\sin\delta \right)}$ value indicates a greater increase in rutting factor after aging, reflecting a more pronounced degree of aging-induced hardening. Accordingly, this index enables an effective evaluation of the resistance of modified bitumen to high-temperature rheological deterioration under thermo-oxidative and ultraviolet aging conditions.

**Figure 2:**
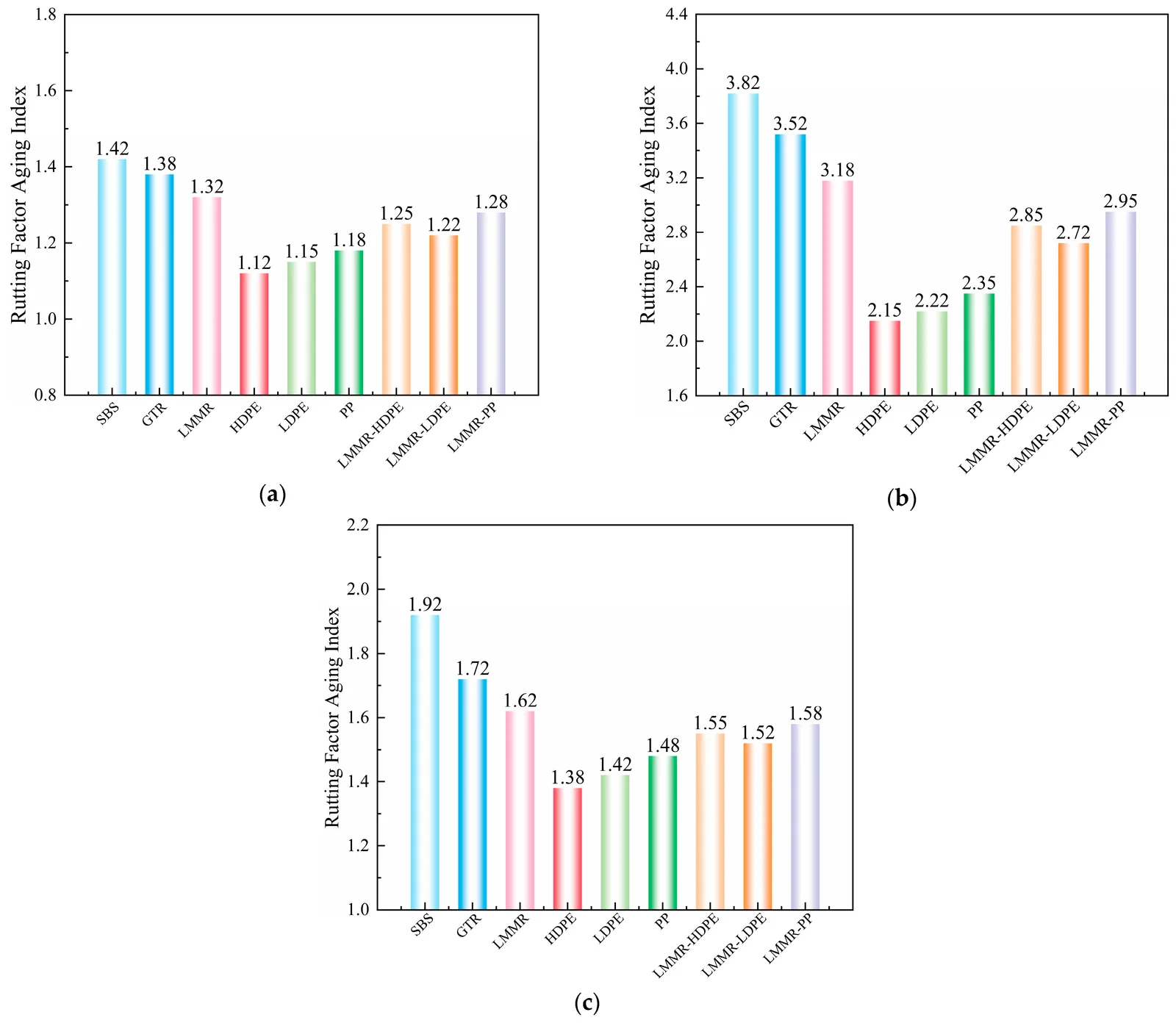
Aging index of rutting factor (G*/sin δ) for different modified bitumen binders after: (**a**) RTFOT aging; (**b**) PAV aging; and (**c**) UV aging.

The aging index of the rutting factor characterizes the variation in the high-temperature rheological properties of modified bitumen before and after aging. A larger index means the aging process makes the material much stiffer. It shows the material is highly sensitive to aging. Figure 2 displays the results for all modified binders after RTFOT, PAV, and UV exposure. Every single aging index is greater than 1.0. This fact proves that all three aging methods raise the high-temperature rheological parameters. Consequently, the binders harden to varying degrees. All sample groups share the exact same trend. Under the selected laboratory protocols, the highest rutting factor aging indices were observed after the RTFOT–PAV sequence. Looking at the specific numbers, the indices stay between 1.12 and 1.42 in Figure 2a. They jump to a higher range of 2.15 to 3.82 in Figure 2b. Then, Figure 2c presents values from 1.38 to 1.92. Comparing the different modifier types, the SBS group generally exhibits a higher aging index, whereas the HDPE group consistently maintains lower levels. Following surface activation, the LMMR group yields lower aging indices than the GTR group; for instance, the value drops from 3.52 to 3.18 under PAV conditions. Moreover, the rubber–plastic-integrated TPE-modified binders generally present lower aging indices than the SBS, GTR, and LMMR groups. Among the three integrated TPE formulations, LMMR–LDPE exhibited the lowest rutting factor aging indices under RTFOT, PAV, and UV conditioning, with values of 1.22, 2.72, and 1.52, respectively, indicating the smallest relative increase in the rutting factor after aging.

The three protocols examine different aspects of binder aging. RTFOT represents short-term thermo-oxidative exposure during binder processing, whereas PAV accelerates oxidative conditioning through elevated temperature and pressurized air. In this study, PAV conditioning was applied to RTFOT-aged residues; the PAV indices referenced to the unaged binders therefore reflect the cumulative RTFOT–PAV history. UV exposure examines irradiation-associated photo-aging of the exposed binder film. The protocols differ in temperature, duration, pressure, and specimen geometry, and equivalent aging doses were not established. Formulation comparisons are therefore made within each protocol. Numerical differences across protocols describe responses to the selected laboratory exposures and do not establish a universal ranking of aging severity.

Examining the various modification systems, both SBS and conventional crumb rubber contain a higher proportion of unsaturated bonds. These structures are highly susceptible to oxidative degradation and cross-linking under the combined effects of heat, oxygen, and ultraviolet radiation, rendering them more sensitive to aging. The lower rutting factor aging indices of LMMR relative to GTR indicate a smaller rheological response to aging under the tested conditions. Improved dispersion and rubber–binder interaction provide a plausible explanation for this difference. For the integrated TPE systems, previous studies have shown that pre-combining rubber and plastic can improve compatibility and promote interacting rubber–plastic phases. Such phase organization could help retain the rheological contribution of the modifier during aging. Ma et al. [32] further reported improved weather-aging resistance of extruded rubber–polyethylene-modified binders, which they associated with polyethylene dispersion and contributions from soluble rubber. The lower rheological aging indices of the present TPE formulations relative to SBS are consistent with this general tendency, although differences in formulation and conditioning preclude direct numerical comparison. Among the three integrated TPE formulations, LMMR–LDPE exhibited the lowest rutting factor aging indices. This behavior may partly reflect the influence of the LDPE molecular architecture on swelling and interactions within the blend, consistent with the dependence of polyethylene phase behavior on branching and crystallinity reported previously.

The preceding analysis demonstrates that while all modified binders experience distinct high-temperature hardening under the three aging conditions, their aging sensitivities differ substantially. Compared to the SBS and conventional crumb rubber-modified binders, the rubber–plastic-integrated TPE-modified bitumen, synthesized via surface activation and reactive extrusion, yields a notably lower rutting factor aging index. Consequently, it exhibits superior resistance to RTFOT, PAV, and UV aging. Among the integrated TPE formulations, LMMR–LDPE exhibited the lowest rutting factor aging indices under each protocol. These results support its comparatively low sensitivity to rheological aging and are consistent with the proposed contribution of rubber–plastic compatibility and phase organization.

### 4.2. Functional Group Aging Index

To evaluate the degree of oxidation of modified bitumen under different aging conditions, the carbonyl index (CI) and the carbonyl functional group aging index (AI_CI_) are employed in this study to characterize the formation and extent of increase in carbonyl oxidation products. They are defined as follows:

(2) $$\mathrm{CI}\;=\;\frac{A_{1700}}{\sum A}$$

(3) $$AI_{\mathrm{CI}}=\frac{CI_{\mathrm{aged}}}{CI_{\mathrm{unaged}}}$$

where A_1700_ is the integrated area of the carbonyl absorption band near 1700 cm^−1^; ∑A is the total integrated area of the characteristic peaks in the range of 600–2000 cm^−1^; and CI_aged_ and CI_unaged_ denote the carbonyl index of the aged and unaged bitumen, respectively. In general, a higher CI value indicates a more pronounced accumulation of carbonyl oxidation products, while a higher AI_CI_ value reflects greater sensitivity of the material to aging.

**Figure 3:**
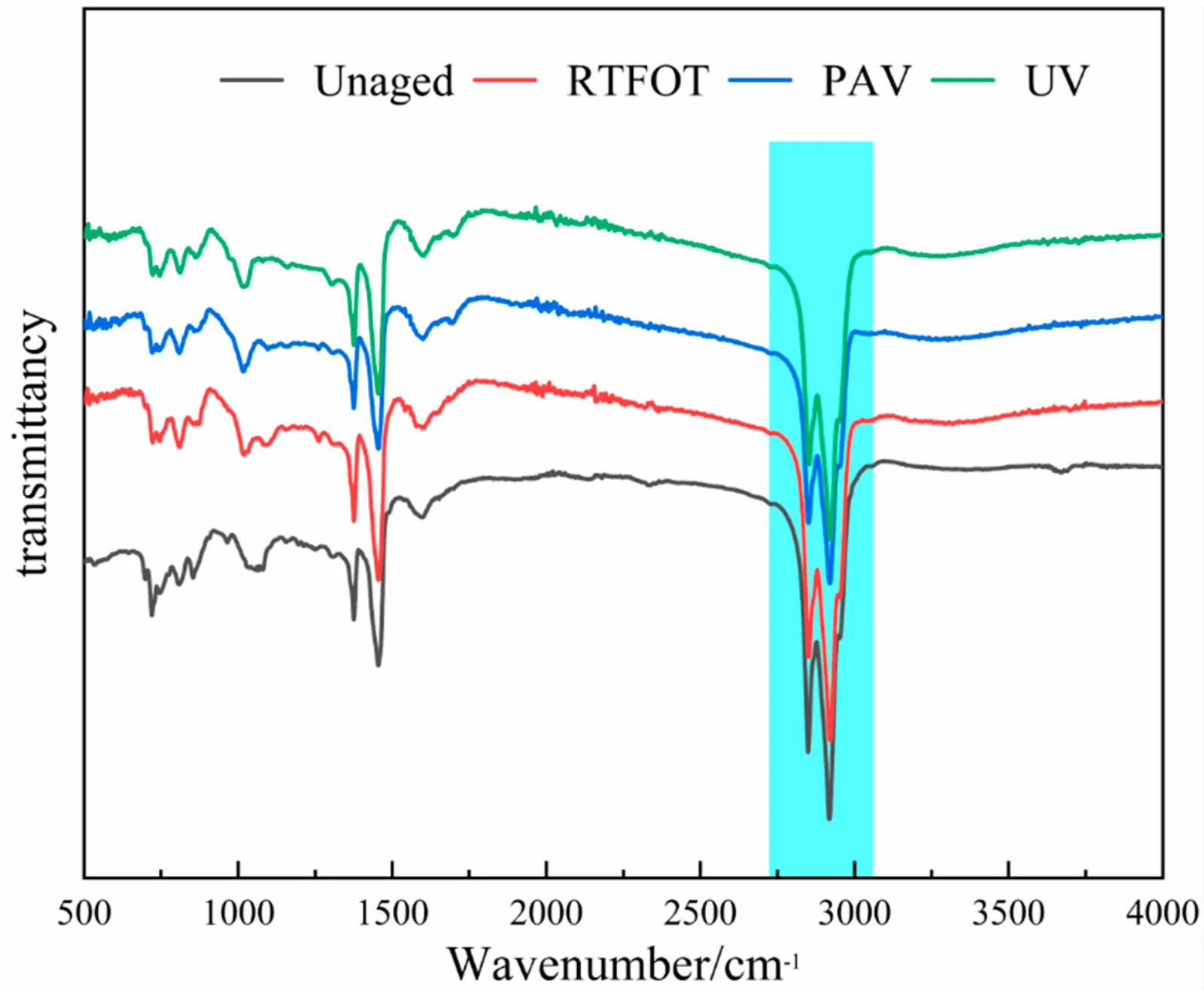
FTIR spectra of HDPE-modified bitumen under different aging conditions.

**Figure 4:**
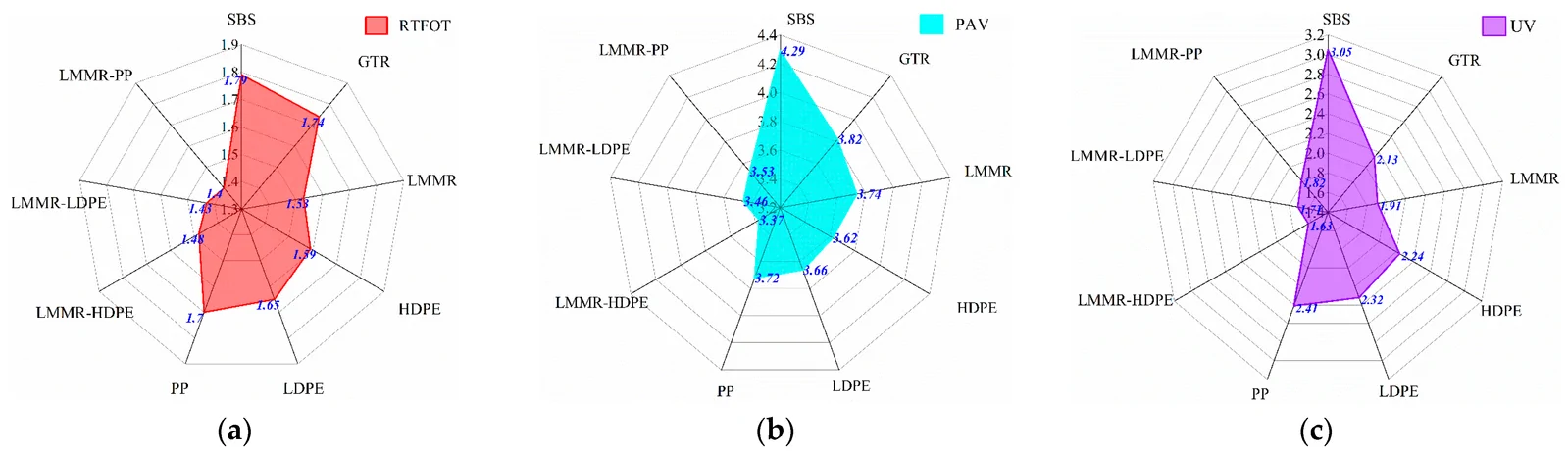
Carbonyl functional group aging index of different types of modified bitumens. (**a**) Carbonyl aging index after RTFOT; (**b**) carbonyl aging index after PAV; (**c**) carbonyl aging index after UV aging.

Figure 3 presents representative FTIR spectra of HDPE-modified bitumen before and after laboratory aging. The main absorption-band positions remained broadly similar across the four conditions. The bands in the 2800–3000 cm^−1^ region correspond to aliphatic C–H stretching vibrations associated with both bitumen constituents and polyethylene. The visual differences in this region are insufficient to establish relative aging severity or specific degradation pathways. Chemical aging is therefore evaluated using the carbonyl index calculated from the integrated absorption near 1700 cm^−1^. The carbonyl index values are reported in Table 8, and the corresponding aging indices are presented in Figure 4.

As shown in Table 8, the carbonyl index (CI) of all modified bitumens increased after aging relative to the unaged samples, confirming that RTFOT, PAV, and UV aging all promoted the formation of carbonyl oxidation products. The highest CI values were measured after RTFOT–PAV conditioning under the selected laboratory conditions. Taking HDPE as an example, its CI rose from 0.0266 in the unaged state to 0.0423 after RTFOT, 0.0963 after PAV, and 0.0596 after UV aging. With respect to the different modification systems, SBS and GTR exhibited CI values of 0.1347 and 0.1297 after PAV aging, respectively, which were higher than those of the other systems, reflecting their greater oxidation susceptibility. In contrast, LMMR-HDPE, LMMR-LDPE, and LMMR-PP consistently displayed lower CI values under all three aging conditions; notably, after PAV aging, their CI values were 0.0923, 0.0913, and 0.0883, respectively, all lower than those of the neat LMMR system. These results indicate that compounding polyolefins with LMMR can effectively inhibit the accumulation of carbonyl oxidation products.

As shown in Figure 4a–c, the carbonyl functional group aging index (AI_CI_) of the different modified bitumens showed differences across the three aging conditions. After RTFOT aging, the AI_CI_ values of SBS and GTR were 1.79 and 1.74, respectively, which were higher than those of LMMR and the rubber/plastic composite systems, suggesting that the conventional elastomer and crumb rubber systems are more susceptible to short-term thermo-oxidative oxidation. Following PAV aging, the AI_CI_ of all systems increased considerably; SBS displayed the highest value of 4.29, while GTR and LMMR reached 3.82 and 3.74, respectively, indicating that long-term thermo-oxidative aging markedly promoted the formation of carbonyl oxidation products. In contrast, the AI_CI_ values of LMMR-HDPE, LMMR-LDPE, and LMMR-PP were 3.37, 3.46, and 3.53, respectively, all lower than those of neat LMMR and the corresponding polyolefin systems. After UV aging, SBS still exhibited the highest AI_CI_ of 3.05, whereas the LMMR-polyolefin composite systems showed values of only 1.63–1.82, demonstrating their stronger inhibitory effect on UV-induced carbonyl growth. Overall, the extent of carbonyl increase ranked in the order: PAV aging > UV aging > RTFOT aging, and the LMMR-polyolefin composite systems consistently displayed relatively low chemical aging susceptibility under all aging environments.

The concurrent increases in the carbonyl index and rutting factor indicate that chemical oxidation accompanied rheological hardening under the tested conditions. Chen et al. [37] similarly found that carbonyl indices increased during extended PAV aging of SBS/rubber-modified binders and were related to selected rheological indices. The different rankings of the present TPE formulations based on rheological and carbonyl aging indices nevertheless show that these measurements capture related but distinct aspects of the aging response. The lower carbonyl growth of the LMMR–polyolefin composites can be interpreted in terms of both chemical susceptibility and phase organization. Previous studies have reported improved compatibility in pre-combined rubber–plastic binders and identified polymer dispersion as a contributor to the aging resistance of rubber–polyethylene composites. On this basis, improved dispersion and interfacial continuity provide a plausible explanation for the present results. A more integrated rubber–plastic arrangement could reduce local interfacial discontinuities and influence oxygen access to oxidation-susceptible regions, thereby contributing to the smaller carbonyl increases. This interpretation links the chemical aging response to the proposed composite morphology without assigning the observed carbonyl changes to a uniquely established mechanism.

### 4.3. Roughness Aging Index

To quantitatively evaluate the degree of surface relief of modified bitumen before and after aging, the root-mean-square roughness, denoted as R_q_, is selected in this study as the AFM morphological parameter. R_q_ reflects the dispersion of height values relative to the mean height within the scanned area; a larger R_q_ value indicates more pronounced local surface undulations and a more heterogeneous phase structure. It is defined as follows:

(4) $$R_{q}=\sqrt{\frac{1}{N}\sum_{i=1}^{N}(z_{i}-\overset{ˉ}{z}{)}^{2}}$$

where N is the total number of sampling points within the scanned area, z_i_ is the height value at the i-th sampling point, and $\overset{ˉ}{z}$ is the mean height over the scanned area. To further compare the effects of different aging regimes on the surface morphology, a roughness aging index, denoted as $AI_{R_{q}}$, is introduced in this study and calculated as:

(5) $$AI_{\left(R_{q}\right)}\;=\;\frac{R_{q,\mathrm{Aged}}}{R_{q,\mathrm{Unaged}}}$$

where R_q,aged_ and R_q,unaged_ represent the root-mean-square roughness of the aged and unaged modified bitumen, respectively. In general, an $AI_{R_{q}}$ value greater than 1 indicates enhanced surface undulation and microstructural coarsening after aging; a higher $AI_{R_{q}}$ corresponds to more pronounced aging-induced morphological deterioration.

All AFM height images were processed in a consistent manner using Nanoscope Analysis 3.0. The raw two-dimensional morphology images were first corrected by the Flatten function to remove background artifacts caused by sample inclination, scanner drift, and baseline curvature. After this correction, the adjusted two-dimensional topography maps could better reflect the intrinsic surface features of the bitumen samples rather than external measurement disturbances. Based on the corrected height data, three-dimensional morphology maps were subsequently reconstructed under the same scanning scale, allowing the surface undulation and local height variation to be visually compared among different samples. Finally, the R_q_ value was extracted from the processed height matrix to quantify the surface roughness. Applying the same processing procedure to all AFM images reduces errors caused by inconsistent image correction and improves the reliability and comparability of roughness parameters among different modified bitumens and aging conditions.

**Figure 5:**
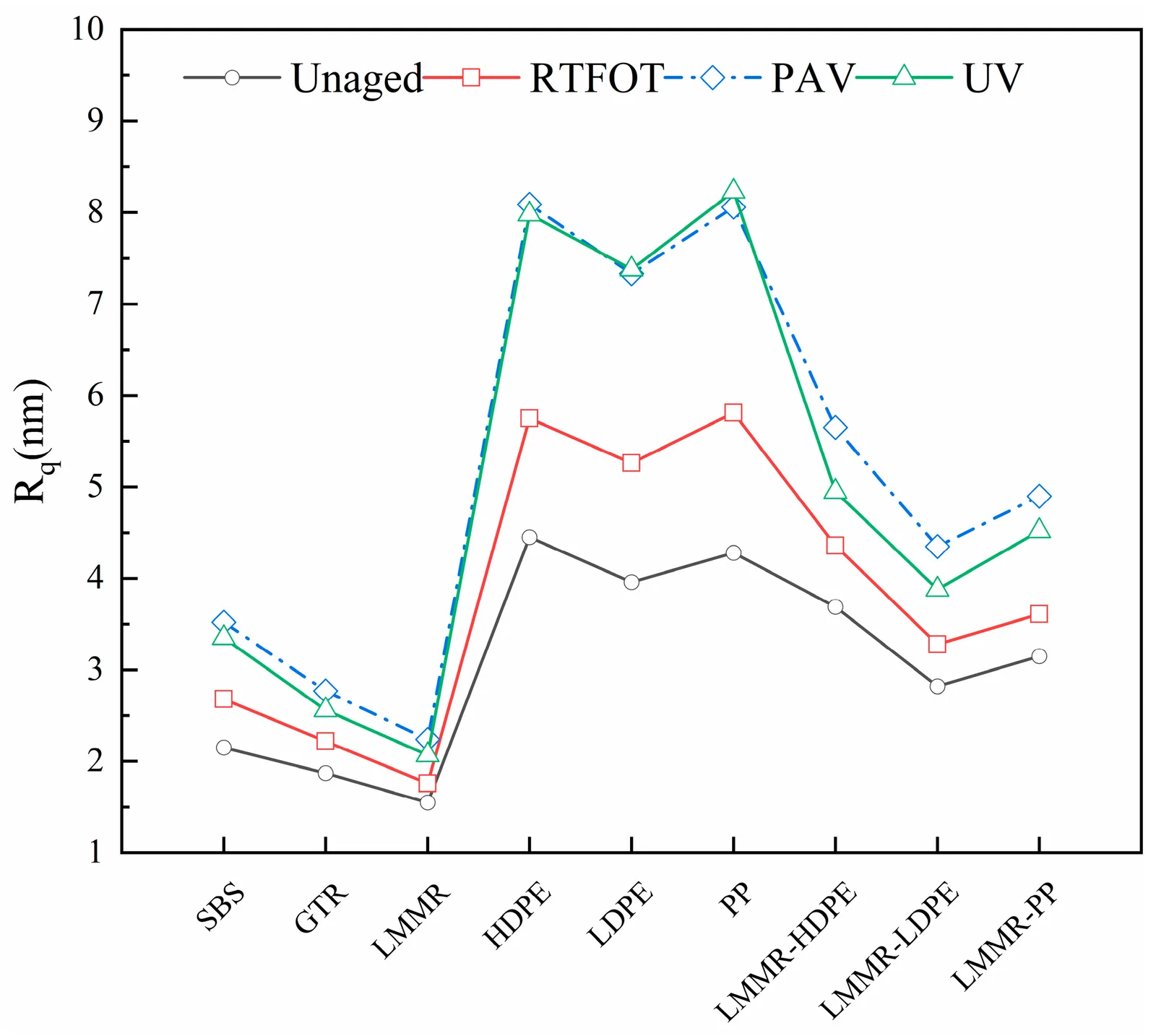
AFM-derived root-mean-square roughness (R_q_) of modified bitumen binders in the unaged state and after RTFOT, sequential RTFOT–PAV, and UV aging.

As presented in Figure 5, the R_q_ values of all modified bitumens increased after RTFOT, PAV, and UV aging compared with their unaged states, indicating that aging intensified surface height heterogeneity and promoted microstructural coarsening. This increase suggests that oxidative and photo-oxidative aging not only alters the chemical composition of bitumen but also disturbs its nanoscale morphological stability. The neat polyolefin-modified bitumens generally exhibited relatively high R_q_ values, with HDPE, LDPE, and PP showing more pronounced surface relief after aging. This behavior can be associated with the local enrichment of polyolefin crystalline domains, insufficient compatibility with the bitumen matrix, and aging-induced phase coarsening. In contrast, the SBS, GTR, and LMMR systems showed lower R_q_ values, implying that the rubber phase provided a certain plasticizing and morphology-regulating effect. Moreover, the LMMR–polyolefin composite systems presented lower R_q_ values than their corresponding neat polyolefin systems, confirming that LMMR reduced crystalline protrusions and phase separation, thereby improving microstructural uniformity.

**Figure 6:**
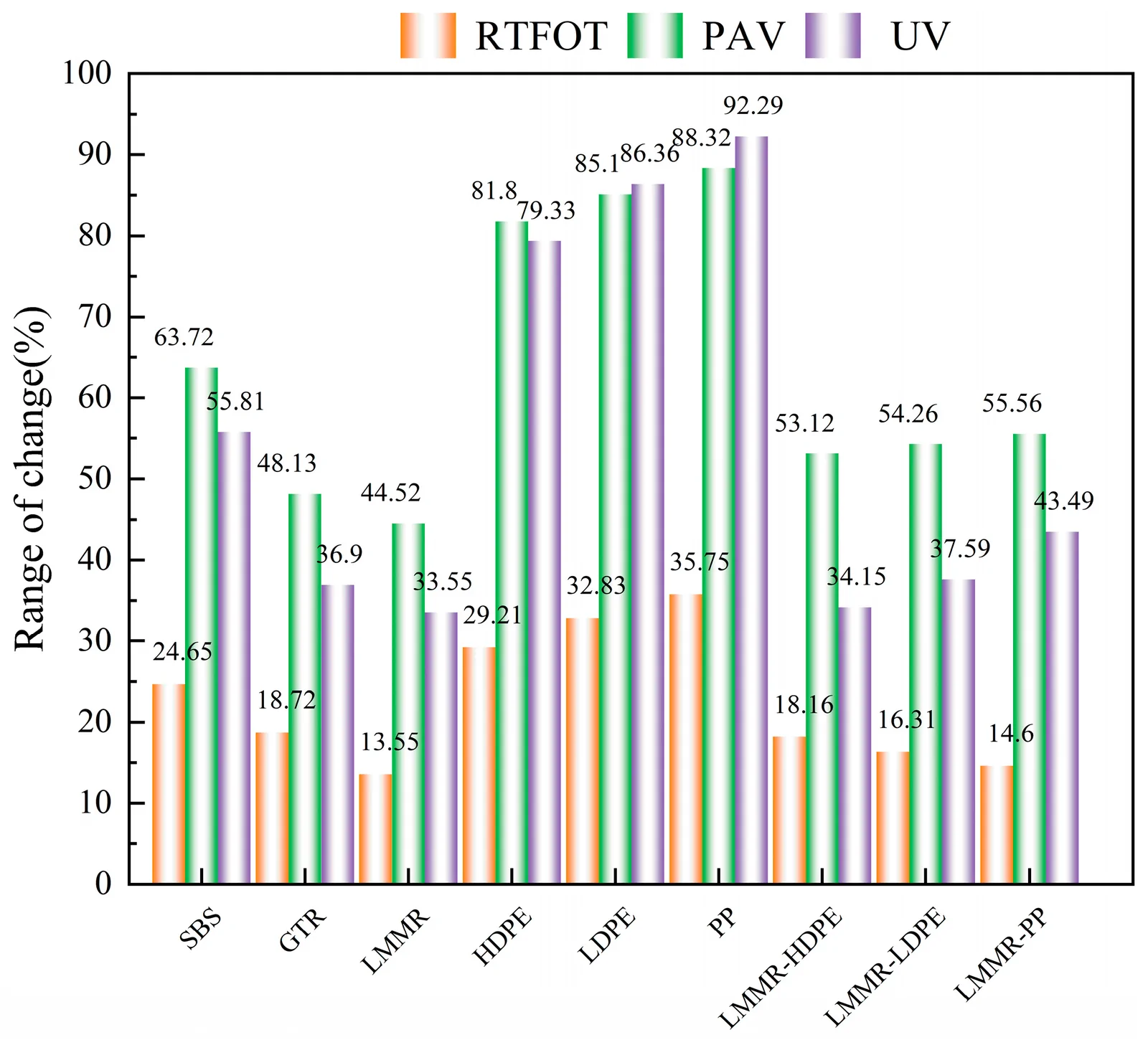
Percentage change in AFM-derived surface roughness (R_q_) of modified bitumen binders induced by RTFOT, PAV, and UV aging.

Figure 6 further illustrates the morphological sensitivity of the different systems to aging. The increase in roughness after RTFOT aging was smaller than that after PAV and UV aging under the tested conditions. In contrast, PAV and UV aging produced larger increases in R_q_, particularly in the neat polyolefin-modified binders. Specifically, HDPE, LDPE, and PP exhibited roughness increases of 81.80%, 85.63%, and 88.32%, respectively, after PAV aging, and remained at high levels ranging from 79.33% to 92.29% after UV aging. These findings suggest that long-term thermo-oxidative and UV exposure promote the coarsening of polyolefin domains, bitumen aggregation, and increased interfacial discontinuity. By comparison, LMMR-HDPE, LMMR-LDPE, and LMMR-PP exhibited lower roughness increases than the corresponding neat polyolefin systems under RTFOT, PAV, and UV aging, demonstrating that the rubber–plastic composite structure can mitigate the extent of aging-induced surface coarsening.

The AFM results complement the FTIR and DSR measurements by describing the topographic response to aging. Xie et al. [40] reported decreases in surface roughness after PAV and UV aging of GO/SBS-modified asphalt, contrasting with the increases in R_q_ observed here. This difference may reflect variations in binder formulation, conditioning history, and AFM evaluation conditions, indicating that the direction of the topographic response should be interpreted within the specific experimental system. Relative to the corresponding neat polyolefin-modified binders, the LMMR–polyolefin composites exhibited lower roughness values and smaller aging-induced roughness increases. Improved integration of the rubber and plastic phases offers a plausible explanation: a more continuous phase arrangement could limit the development of pronounced local height differences during aging. Notably, LMMR–LDPE showed the lowest rheological aging indices among the integrated TPEs, whereas LMMR–HDPE exhibited lower carbonyl and roughness aging indices after PAV and UV conditioning. These different rankings suggest that carbonyl formation and surface roughness each capture only part of the overall aging response. The three techniques therefore provide complementary information, with interfacial stabilization serving as a possible explanation for the formulation-dependent behavior.

### 4.4. Multi-Index Evaluation of the Aging Resistance of Rubber–Plastic-Based Integrated TPE-Modified Bitumen with SBS as the Benchmark

SBS-modified bitumen was used as the reference for evaluating the relative aging responses of the three TPE-modified binders. For each aging protocol, the rutting factor aging index, carbonyl aging index, and percentage change in R_q_ were normalized separately to the corresponding SBS values. A normalized value below 1.0 indicates a lower response for that particular indicator than the SBS reference under the same protocol. As shown in Table 9, Table 10 and Table 11, the normalized values of the three TPE systems were below 1.0. This normalization facilitates comparisons with SBS within each response parameter and aging protocol; it does not establish a common scale of aging severity across the three protocols.

As shown in Table 9, Table 10 and Table 11, all RA values of the three composite modified bitumen groups (LMMR-HDPE, LMMR-LDPE and LMMR-PP) under the three aging conditions are less than 1.0. By comparing the three categories of indices, it is found that the overall reduction amplitude of RAI in terms of chemical oxidation (AI_CI_) and micromorphology ($AI_{\left(R_{q}\right)}$) of the three groups of materials is larger than that of rheological property ($AI_{\left(G^{*}/\sin\delta \right)}$), and the reduction in RAI is more significant, especially under long-term pressure aging (PAV) and ultraviolet photo-oxidative aging (UV) conditions. This indicates that the rubber–plastic-based integrated TPE system has a more prominent synergistic retardation advantage in inhibiting the accumulation of oxidation products and delaying the deterioration of surface microstructure.

**Figure 7:**
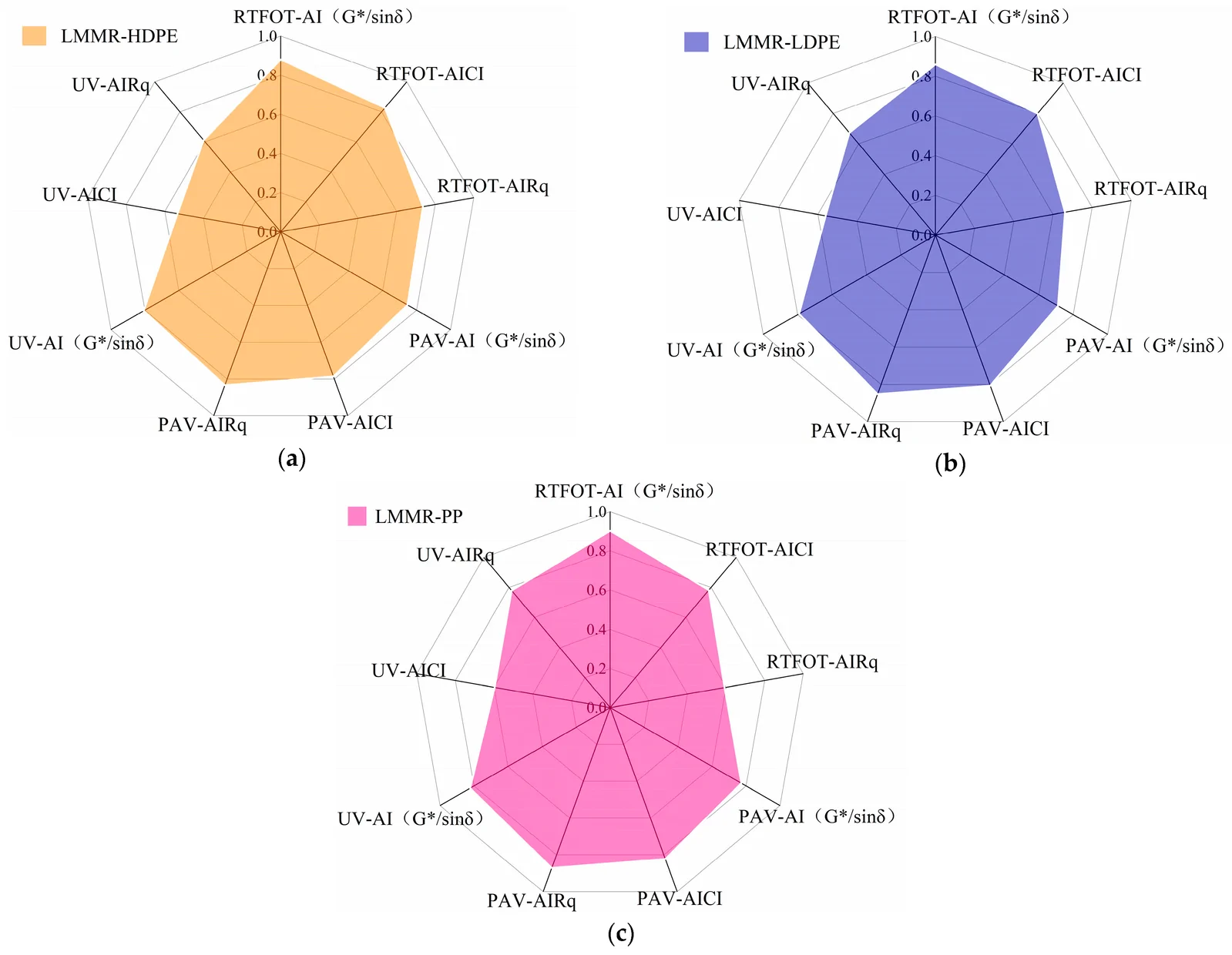
SBS-normalized aging-response indices of integrated TPE-modified bitumen: (**a**) LMMR–HDPE; (**b**) LMMR–LDPE; and (**c**) LMMR–PP. The rutting factor aging index, carbonyl aging index, and relative increase in R_q_ are normalized separately to the corresponding SBS values under the same aging protocol. All plotted values are dimensionless.

As shown in Figure 7, all nine relative ratio values of the three rubber–plastic-based integrated TPE-modified bitumen are below the SBS benchmark value of 1.0, while the focus of performance advantages differs among different systems. The LMMR-LDPE group exhibits superior performance in high-temperature rheological aging resistance, with $AI_{\left(G^{*}/\sin\delta \right)}$ values of 0.8592, 0.7120 and 0.7917 under RTFOT, PAV and UV conditions, respectively, all of which are the lowest among the three groups, indicating its greater advantage in maintaining high-temperature rheological stability. The LMMR-HDPE group presents better performance in chemical oxidation stability and microstructure stability, with PAV-AI_CI_, UV-AI_CI_, PAV-$AI_{\left(R_{q}\right)}$ and UV-$AI_{\left(R_{q}\right)}$ values of 0.7855, 0.5344, 0.8336 and 0.6119, respectively, all lower than those of the other two groups, which demonstrates that it has a stronger inhibitory effect on the oxidation and roughening evolution induced by PAV and UV aging. The LMMR-PP group shows good performance under RTFOT conditions, with RTFOT-AI_CI_ and RTFOT-$AI_{\left(R_{q}\right)}$ of 0.7821 and 0.5923, respectively, which are the lowest among the three groups, while the relevant indexes are relatively high under PAV and UV aging conditions, indicating that its anti-aging advantage is mainly reflected in the short-term aging stage.

**Figure 8:**
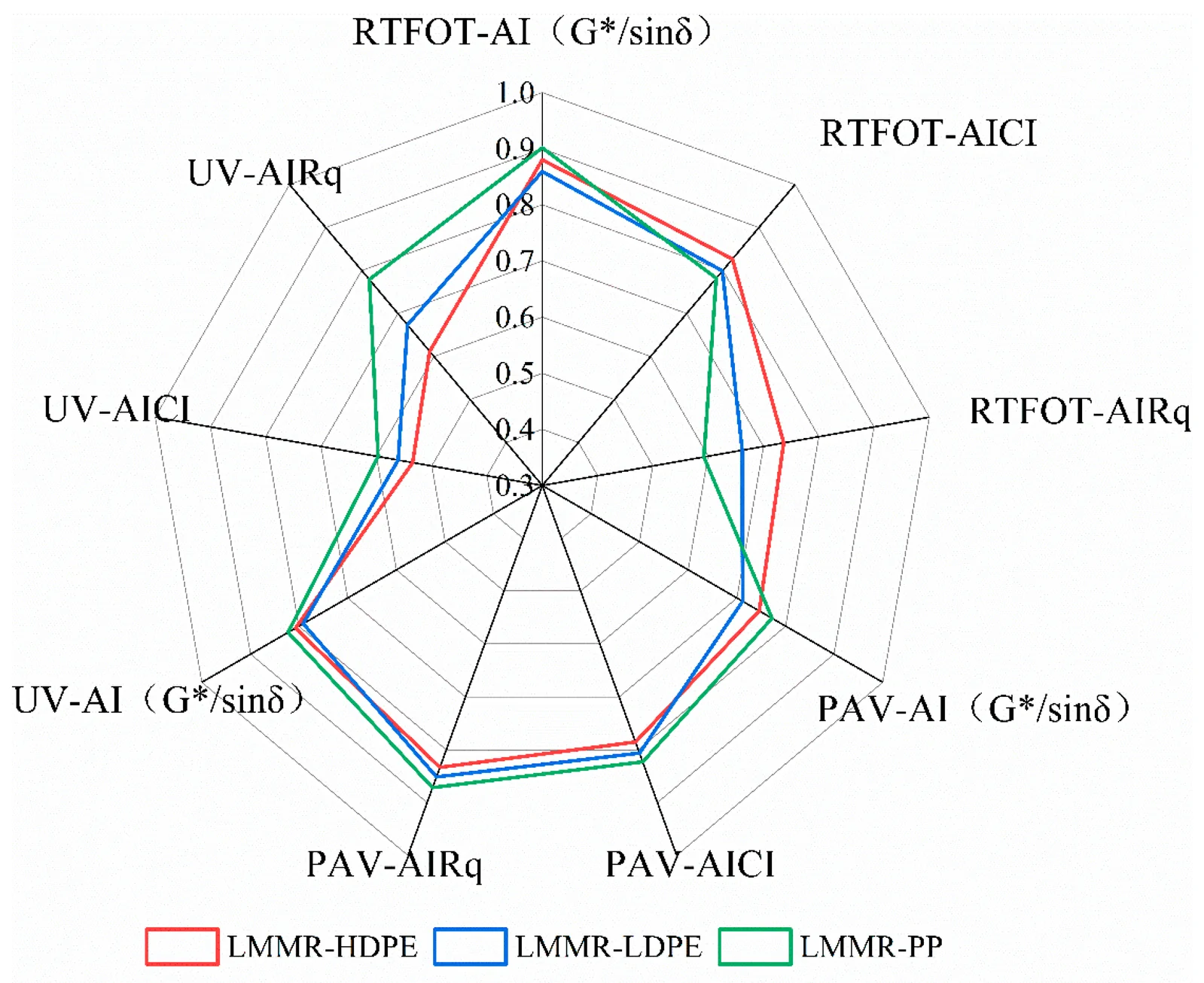
Comparison of SBS-normalized aging-response indices among the three integrated TPE formulations. All plotted values are dimensionless, with normalization performed separately for each indicator and aging protocol using the corresponding SBS value.

As further illustrated in Figure 8, the LMMR-LDPE system is located closer to the center of the radar chart along the three $AI_{G^{*}/\sin\delta }$ axes, indicating its stronger ability to retain high-temperature rheological stability after different aging treatments. In comparison, the LMMR-HDPE system shows a more evident contraction along the PAV-AI_CI_, UV-AI_CI_, PAV-$AI_{R_{q}}$, and UV-$AI_{R_{q}}$ directions, suggesting that HDPE is more effective in suppressing oxidative functional group accumulation and microstructural roughening under severe thermo-oxidative and photo-oxidative conditions. By contrast, the LMMR-PP system exhibits relatively larger outward expansion for the indexes associated with PAV and UV aging, although it still shows certain advantages under RTFOT aging. These results reveal indicator-dependent differences among the three TPE formulations: LMMR–LDPE exhibits the smallest rheological aging response, whereas LMMR–HDPE shows lower carbonyl and roughness aging indices after PAV and UV conditioning. The relative advantages of LMMR–PP are more evident under RTFOT conditioning.

Overall, all three rubber–plastic-based integrated TPE-modified bitumen samples exhibit better comprehensive anti-aging performance than SBS-modified bitumen, with different focuses of performance advantages. The LMMR-LDPE group shows the best performance in high-temperature rheological aging resistance; the LMMR-HDPE group has greater advantages in chemical oxidation stability and microstructure stability, while the LMMR-PP group presents certain advantages under short-term thermo-oxidative aging conditions. It can be concluded that the rubber–plastic-based integrated TPE-modified bitumen can enhance the anti-aging performance of the material at multiple levels, including macroscopic rheology, chemical functional group evolution and micromorphology stability. Among them, the LMMR-HDPE group and LMMR-LDPE group present better comprehensive performance, which can be taken as the key directions for the subsequent optimal design of rubber–plastic-based integrated TPE-modified bitumen.

Compared with the corresponding polyolefin reference binders, the integrated TPE formulations showed indicator-dependent aging responses. Smaller carbonyl and roughness increases were not consistently accompanied by lower rutting factor aging indices. These comparisons concern the complete formulations at the investigated dosages, since the formulations differ in total additive loading and the experimental design does not independently separate the effects of LMMR incorporation, compatibilization, and processing.

### 4.5. Process Reproducibility and Scale-Up Considerations

Process reproducibility requires control of feedstock variability as well as extrusion conditions. Relevant factors include LMMR viscosity and particle size, recycled-polyolefin composition and melt-flow behavior, pretreatment history, and compatibilizer dosage. The formulation, temperature profile, screw speed, and processing sequence reported here provide a basis for reproducing the laboratory route. Transfer to a larger extruder requires attention to screw configuration, mass throughput, residence-time distribution, and actual melt temperature, because equivalent thermal and shear histories cannot be assumed from unchanged barrel-temperature settings and rotational speeds alone. Maintaining adequate dispersion while avoiding excessive polymer degradation is a key challenge. Feed metering, cooling, and pelletization also require consistent control. Pilot-scale trials should assess consistency across feedstock lots and production batches, using modifier uniformity and the rheological and aging responses of the resulting binders as quality criteria.

## 5. Conclusions

In this study, low-Mooney reclaimed rubber (LMMR) derived from waste tires was used as the rubber phase, while HDPE, LDPE, and PP were employed as the plastic phases. SEBS-g-MAH was introduced as a reactive compatibilizer. Rubber–plastic-based integrated TPE modifiers were prepared through the synergistic combination of surface activation and reactive extrusion. Their high-temperature rheological properties, chemical structural evolution, and surface morphology under RTFOT, PAV, and UV aging were systematically evaluated. The main conclusions are as follows:

(1) RTFOT, PAV, and UV conditioning increased the rutting factor, carbonyl index, and surface roughness relative to the unaged binders. These changes represent responses to short-term thermo-oxidative exposure, cumulative RTFOT–PAV conditioning, and UV irradiation, respectively, under the selected laboratory conditions.
(2) Compared with the SBS reference, the integrated TPE-modified binders exhibited lower rutting factor, carbonyl, and roughness aging indices under each of the three laboratory protocols. These complementary results indicate smaller aging-related changes in high-temperature rheology, chemical oxidation, and surface topography.
(3) Compared with GTR-modified bitumen, LMMR-modified bitumen exhibited lower rheological aging indices, smaller relative carbonyl increases, and less surface roughening under the tested aging conditions. Improved rubber–binder interaction associated with rubber treatment provides a plausible explanation for these trends.
(4) The relative advantages of the three integrated TPE formulations depended on the aging indicator and protocol. LMMR–LDPE exhibited the lowest rutting factor aging indices under all three protocols. LMMR–HDPE showed the lowest carbonyl aging indices and relative roughness increases after PAV and UV conditioning, whereas LMMR–PP showed the lowest values for these two indicators after RTFOT conditioning. Formulation selection should therefore reflect the targeted rheological, chemical, or morphological aging response.

Overall, the integrated TPE formulations exhibited favorable laboratory aging responses across rheological, chemical, and topographic indicators, supporting the potential of the combined surface-treatment and extrusion-based preparation route for modified bitumen. This approach provides a feasible strategy for the synergistic and high-value utilization of waste tire rubber and recycled plastics in durable bitumen materials.

### Limitations and Future Research

The findings are restricted to the formulations and laboratory aging conditions examined in this study, which do not fully reproduce the complexity of field exposure. The comparisons are descriptive and do not establish statistical significance or a universally optimal formulation. Future work should quantify experimental variability using independently prepared replicate specimens, evaluate mixture-level rutting, cracking, fatigue, and moisture resistance, and validate long-term performance under field conditions.

## Figures and Tables

**Table 1:** Basic properties of Donghai-70# base bitumen.

Properties	Measured Value	Specification	Test Method
Penetration at 25 °C (0.1 mm)	68	60–80	T0604
Softening point (°C)	49.5	≥46	T0606
Ductility at 10 °C (cm)	24.5	≥20	T0605
Rotational viscosity at 135 °C (Pa·s)	0.41	≤3.0	T0625
Density at 15 °C (g/cm^3^)	1.021	Reported	T0603

**Table 2:** Technical properties of road-grade linear SBS modifier.

Properties	Measured Value
Styrene content (%)	31
Block ratio (S/B)	30/70
Melt flow rate at 200 °C under a 5 kg load (g/10 min)	2.5
Appearance	White pellets

**Table 3:** Technical properties of GTR and LMMR.

Component	GTR	LMMR
Natural rubber (%)	43.32	46.51
Synthetic rubber (%)	15.92	18.50
Sol content (%)	42.50	56.52
Inorganic filler (%)	6.87	6.81
Carbon black (%)	33.91	28.23

**Table 4:** Technical indicators of recycled plastics.

Polymer Type	Appearance	Density (g/cm^3^)	Melting Point (°C)	Crystallinity (%)
HDPE	Flat pellets	0.940~0.961	142	70~90
PP	Flat pellets	0.900~0.910	176	50~70
LDPE	Flat pellets	0.910~0.925	110	10~30

**Table 5:** Technical properties of SEBS-g-MAH compatibilizer.

Grade	Density (g/cm^3^)	Viscosity (cP)	MAH Content (%)	Appearance	Styrene/Rubber Ratio
FG1901	0.91	1000	1.4~2	Transparent pellets	30/70

**Table 6:** Formulations of rubber–plastic-based integrated TPEs.

Rubber–Plastic-Based TPE	Formulation
LMMR-HDPE TPE	70% LMMR + 30% HDPE + 5% SEBS-g-MAH
LMMR-LDPE TPE	70% LMMR + 30% LDPE + 5% SEBS-g-MAH
LMMR-PP TPE	70% LMMR + 30% PP + 5% SEBS-g-MAH

**Table 7:** Formulations of different modified bitumens.

Abbreviation	Formulation
SBS	4.0% linear SBS + base bitumen
GTR	10.5% GTR + base bitumen
LMMR	10.5% LMMR + base bitumen
HDPE	4.5% HDPE + base bitumen
LDPE	4.5% LDPE + base bitumen
PP	4.5% PP + base bitumen
LMMR-HDPE	15% LMMR-HDPE TPE + base bitumen
LMMR-LDPE	15% LMMR-LDPE TPE + base bitumen
LMMR-PP	15% LMMR-PP TPE + base bitumen

**Table 8:** Carbonyl index of modified bitumen binders at unaged and aged states.

Modified Bitumen Type	Unaged	RTFOT	PAV	UV
SBS	0.0314	0.0562	0.1347	0.0958
GTR	0.0339	0.059	0.1297	0.0722
LMMR	0.0292	0.0447	0.1092	0.0558
HDPE	0.0266	0.0423	0.0963	0.0596
LDPE	0.0255	0.0421	0.0933	0.0592
PP	0.0249	0.0423	0.0926	0.06
LMMR-HDPE	0.0274	0.0406	0.0923	0.0447
LMMR-LDPE	0.0264	0.0378	0.0913	0.0451
LMMR-PP	0.025	0.035	0.0883	0.0455

**Table 9:** Relative anti-aging performance of different modified bitumens after RTFOT.

Modified Bitumen	$AI_{\left(G^{*}/\sin\delta \right)}$	AI_CI_	$AI_{\left(R_{q}\right)}$
SBS	1	1	1
LMMR-HDPE	0.8803	0.8268	0.7367
LMMR-LDPE	0.8592	0.7989	0.6617
LMMR-PP	0.9014	0.7821	0.5923

**Table 10:** Relative anti-aging performance of different modified bitumens after PAV.

Modified Bitumen	$AI_{\left(G^{*}/\sin\delta \right)}$	AI_CI_	$AI_{\left(R_{q}\right)}$
SBS	1	1	1
LMMR-HDPE	0.7461	0.7855	0.8336
LMMR-LDPE	0.712	0.8065	0.8515
LMMR-PP	0.7723	0.8228	0.8719

**Table 11:** Relative anti-aging performance of different modified bitumens after UV aging.

Modified Bitumen	$AI_{\left(G^{*}/\sin\delta \right)}$	AI_CI_	$AI_{\left(R_{q}\right)}$
SBS	1	1	1
LMMR-HDPE	0.8073	0.5344	0.6119
LMMR-LDPE	0.7917	0.5607	0.6735
LMMR-PP	0.8229	0.5967	0.7793

## Data Availability

The original contributions presented in this study are included in the article. Further inquiries can be directed to the corresponding authors.
